# Preparation of photoactive ZnS-composite porous polymer films: Fluorescent and morphological properties

**DOI:** 10.1080/15685551.2021.1989151

**Published:** 2021-10-13

**Authors:** Guadalupe Del C. Pizarro, Wilson Alavia, Rudy Martin-Trasanco, Oscar G. Marambio, Julio Sánchez, Diego P. Oyarzún

**Affiliations:** a Departamento De Química, Facultad De Ciencias Naturales, Matemáticas Y Medio Ambiente, Universidad Tecnológica Metropolitana, Santiago, Chile; b Programa Institucional De Fomento a La Investigación, Desarrollo E Innovación (Pidi), Universidad Tecnológica Metropolitana, Santiago, Chile; c Universidad de Santiago de Chile (USACH), Facultad de Química y Biología, Departamento de Ciencias del Ambiente, Santiago, Chile; d Departamento de Química y Biología, Facultad de Ciencias Naturales, Universidad de Atacama, Copiapó, Chile

**Keywords:** Stimulus-responsive porous polymer films, porous structured films, morphological surface characteristic, light-sensitive materials, spiropyrans

## Abstract

This work describes the use of the breath figure (BF) method for the fabrication of photoactive porous polymer films and the characterization of their responsive to photo stimulus. The films incorporate self-assembled photoactive polymers and ZnS nanoparticles (NPs). The effect of both components on the optical and morphological properties of the films were analyzed. Films with a hexagonally ordered pattern were obtained. The photoactive polymer was prepared by grafting the photochromic component 1-(2-hydroxyethyl)-3,3-dimethylindoline-6-nitrobenzopyran (*SP*) to polystyrene-*block*-polymethacrylic acid (PS-*b*-PMMA). ZnS NPs were incorporated into the polymer solution, and the films were prepared using spin-coating on glass substrates before subjecting them to the BF method. The hollow footprints were obtained before introducing the ZnS NPs in order to maintain the necessary conditions for hexagonal film growth. Accordingly, the SEM micrographs of the films prepared in the presence of ZnS NPs displayed a loss in the pore arrangement as a consequence of the interaction between *SP* moiety and NPs. The light-emitting properties of films were characterized by blue and violet colors when exposed to UV light under fluorescence. Progress in the field of breath-figure formation and its application, such as exemplified in this work, leads to functional structures with suitable applications in chemistry and materials science. It is expected that such microstructured polymeric films will have interesting applications in photonic and optoelectronic devices.

## 1. Introduction

The search for a variety of new functional materials, such as films with highly regular porous patterning has been received great interest due to their potential use in material technology [1,2].

A dynamic breath figures (BFs) templating method for polymers [3–6] and other materials is an attractive method to prepare such films. Through the BF method, condensed water can create periodic structures with a size range between 50 nm and 20 µm. Films with smaller pores are used to study energy transfer and hold promise in potential photovoltaic applications, while films with larger pores are attractive as templates for cell growth and as microlens arrays [7].

Moreover, porous films might find application in membranes [8], in photonic [9] and/or opto-electronic devices [10]. Regarding to the latter two, the exploration of light-responsive molecules in devices typically requires their immobilization on a surface through a linker that does not interfere with the structures’ light switching behaviour. This has been achieved for photoswitchable molecules by the formation of self-assembled monolayers (SAMs) [11], and bilayers, and also by the incorporation of SAMs onto polymer films [12,13] and polymer beads. The reversibility of these macroscopic properties is a result of photoinduced transformations at the molecular level [14,15]. These processes can be explored with the aim of tuning the optical signals and thus offer the opportunity to design and implement photonic devices for optical processing based on molecular components [15]. The change produced in the chemical structure allows it to absorb in a region of the spectrum, generally in the visible, after which, a second radiation or thermal stimulus returns it to its basal state [16,17]. In fact, the terms ‘positive’ and ‘negative’ photochromism are generally used to indicate photoinduced coloration and decoloration processes, respectively. However, both transformations must be reversible by definition [17].

Spiropyrans (*SP*), reported first by Fischer and Hirshberg in 1962 [18], are one of the most widely studied classes of photoswitchable compounds. The irradiation of *SP* compounds with near-UV light [19] or its electro-oxidation [20] induces the heterolytic cleavage of the spiro carbon-oxygen bond, which leads to its ring-opening and converting into merocyanine (*MC*). The intense absorption in the visible region of the open-form *MC* has led to the advanced study of *SP* compounds in photochromic [16], molecular optoelectronic [21] optobioelectronic fields [22], and chemical sensing [23].

It is known that the presence of semiconductor nanoparticles linked to the photochromic agent modulates the absorbance and/or redox potentials of the former. Additionally, the optical emission of these NPs can not only be modulated but also activated/deactivated in a switching-on/off molecular fashion [24,25]. Thus, these clever operating principles for fluorescence modulation can complement the *SP* to *MC* photoconversion in nanostructured organic materials, leading to the development of innovative functional material composites [24–27]

Previously, a series of hybrid polymer films and their optical properties were reported by our research group. In these previous works, fluorescent ZnS NPs at different wt % were inserted into the polymer matrices in order to modify their optical and thermal properties. As a result, significant effects on the optical properties of the hybrid materials were also observed [28–30].

In this work, a series of porous photo switchable films containing both, *SP* and ZnS NPs, were obtained and their photoluminescent properties at different compositions of photochromic agent and template processing parameters were studied. Moreover, due to the interplay between both photoactive components unique and tuneable fluorescent properties was observed. The morphological and fluorescent properties due to the effect of UV irradiation were studied using fluorescence microscopy and SEM. This work aims to provide a valuable contribution to the design and implementation of photonic devices for optical processing based on molecular and macromolecular components.

## 2. Experimental part

### Characterization

The absorption spectra of the films were recorded at 25°C between 250 and 700 nm using a Perkin Elmer Lambda 35 spectrophotometer. The FT-IR spectra were recorded on a Perkin-Elmer Spectrum-Two spectrometer with an UATR unit coupled in the range 4000–500 cm^−1^ with a resolution of 1 cm^−1^. Photoluminescence (PL) measurements were performed at room temperature by a fluorescence spectrometer system (Perkin Elmer, model L 55). The number averages (*M*_n_), weight average (*M*_w_) molecular weights, and polydispersity (*M*_w_/*M*_n_) of the polymers were determined by size exclusion chromatography (SEC) using a Shimatzu LC 20 instrument equipped with RI detector. The morphological properties of the photoactive block copolymer films were examined using SEM and optical fluorescence microscopy. The SEM was a model LEO 1420VP with a 100 μA beam current and a working distance of 12–14 mm. The microscope was operated at high vacuum (system vacuum ~10^−6^ mbar and chamber 10^−3^ mbar). An optical microscopy LEICA Model DM2000 LED with a camera LEICA MFC 170 HD was used. The camera was set to an automatic exposure of 500.00 ms, saturation of 120 and gamma 0.00. The image surface was 549.45 μm x 412.09 μm. For image acquisition under fluorescence, the aperture used was 1/3 and the focus 3/3.

The films were prepared using the BF methodology with a chamber Darwin model PH9-DA with relative humidity (RH) control of 75% at 25°C. X-ray diffraction analysis was performed at room temperature on a Bruker D-8 Advanced Diffractometer, using a long, fine-focus ceramic X-ray tube with a copper anode (k(CuKa) = 1.54 A°) working at 2.2 kW. The diffraction patterns were obtained in the usual Bragg–Brentano (h-2 h) geometry via powder samples in a rotating holder. The X-ray tube was operated at 40 kV and 40 mA.

## Synthesis and Characterization of the photoactive polymer

The photoactive polymer was obtained using the experimental procedure described elsewhere [31–33]. PS-*b*-PMMA was obtained with a narrow polydispersity index (D = 1.2), and weight average (*M*_w_) molecular weights of 24.8 kDa via atom transfer radical polymerization (ATRP). The catalytic system was integrated by bipyridine/Cu(I) complexes, ethyl-2-bromopropionate as initiator, PS macroinitiator, and the MMA monomer in a molar ratio of 1/1/2/100, respectively. The process above was described in more detail in [33]. The copolymer composition percentage for PS/PMMA blocks were PS = 80% and PMMA = 20%. These results were calculated via the ^1^H-NMR spectroscopy using the area from proton signal attributed to -CH_3_ group, (MMA) monomer at 0.8–1.2 ppm, 3 H, and the area of aromatic ring protons from styrene group at 6.3–7.3 ppm, (5 H). Posteriorly, the resulting of photoactive polymers derived from N-(2-methacryoxyethyl)-6-nitro-spirobenzopyranindoline and methyl methacrylate moiety by condensation reaction was prepared. By applying this procedure, the photoactive molecules were incorporated into the main MMA moiety (20%) in a composition percentage of approximately of 80%. Accordingly, the final copolymer composition percentage ratio of PMMA:SP found was 20:80%. It was estimated by the variation in the integration values at 3,7 ppm assigned to the ester groups.

**Figure 1.**
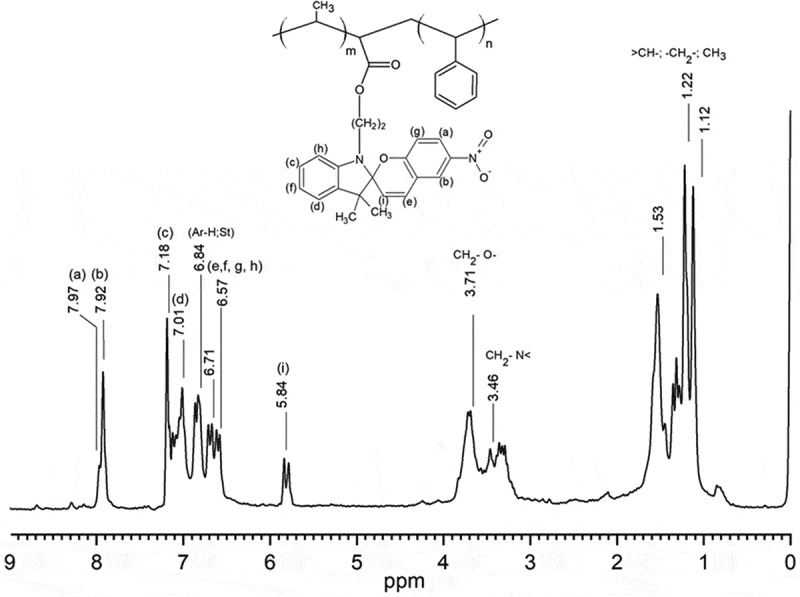
^1^H-NMR (CDCl_3_, 400 MHz) of the photoactive polymer PS-*b*-(MMA)-*SP.*

The ^1^H-NMR (CDCl_3_, 400 MHz) of the photoactive polymer exhibit the following signals: δ 7.8–8.00 (m, 2 H), 7.00–7.20 (m, 2 H), 6.84 (5 H Ar ring of St), 6.71–6.57 (m, 4 H), 5.84 (1H, s), 3.71 (2 H, m), 3.46 (2 H, m), 1.53 (3 H, s from MMA), 1.40–1. 5 (3 H, m), 1.22 (3 H, s), 1.12 (3 H, s), see Figure 1. The FT-IR spectrum of the block copolymer exhibited characteristic absorption bands at 3066–3026 cm^−1^ [υ(CH, aromatic)]; 2923–2852 cm^−1^ [υ(CH, CH_2_)]; 1948–1875 cm^−1^ [υ(aromatic overtone)]; 1730.96 cm^−1^[υ(-C = O from acid)]; 758.31 and 691.26 cm^−1^ [υ(aromatic ring)].

### Preparation of ZnS NPs

The conversion of Zn^2+^ to ZnS was achieved by dropping an equimolar solution of Na_2_S (1.0 g in 10 mL of THF) to the stirring solution. The average crystallite size was calculated as 40–60 nm using Scherrer’s equation. D = kλ /β cos θ where D is the crystallite size, k is a constant (0.9 for the spherical shape), λ is the wavelength of the X-ray radiation, β is the line width obtained after correction for instrumental broadening and θ is the angle of diffraction [29].

### Preparation of photoactive block copolymer/ZnS nanocomposite

The ZnS NPs were incorporated into the photoactive polymer solutions at different concentrations. The polymer was cast on to a glass substrate and then spin-coated (Chemat Scientific, coupled with an oil-free vacuum pump Rocker Chemker 410). The films were further fabricated by subjecting the coated glass to BF methodology under controlled experimental conditions. UV light with a center wavelength of 365 nm was used.

The ATR-FTIR spectra of the prepared films were recorded. The samples showed signals υ (cm^−1^) characteristic of the expected functional groups at 3430 (O-H stretching, carboxylic acid); 1712 (stretching of -C = O, carboxylic acid); 1630 (stretching of -C = O, ester); 1292 and 1177 (symmetric and asymmetric alkyl ester stretching). Typical broad signals from the interaction of the ZnS NPS with the functional groups were observed at 580 cm^−1^ [28].

## 3. Results and discussion

### Characterization by UV-visible spectrophotometry, optical fluorescence microscope and SEM microscopy

**Figure 2.**
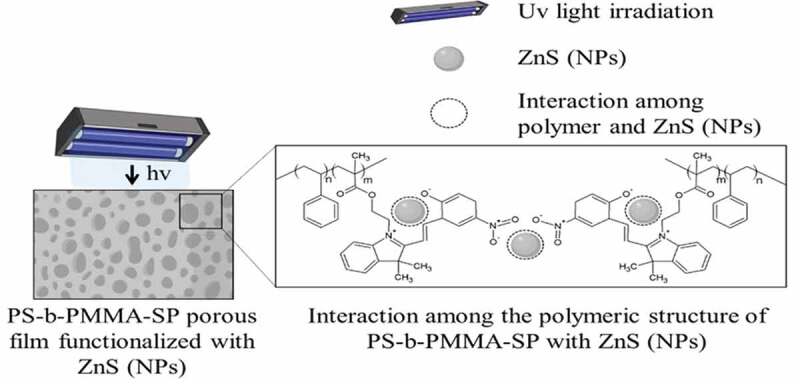
Schematic representation of the polymer films-(*SP*) functionalized with ZnS NPs

The solution of polymer with 1-(2-hydroxyethyl)-3,3-dimethyl indoline-6-nitrobenzopyran (*SP*) moiety showed photoinduced reversible interconversion upon UV-Vis irradiation (365 nm). The photoinduced formation of the colored state ‘merocyanine’ (Figure 2b) is responsible for the significant increases of absorbance in the visible region. As a result, the absorbance of the photochromic solution at 578 nm can be modulated simply by turning an ultraviolet source on and off.

**Figure 3.**
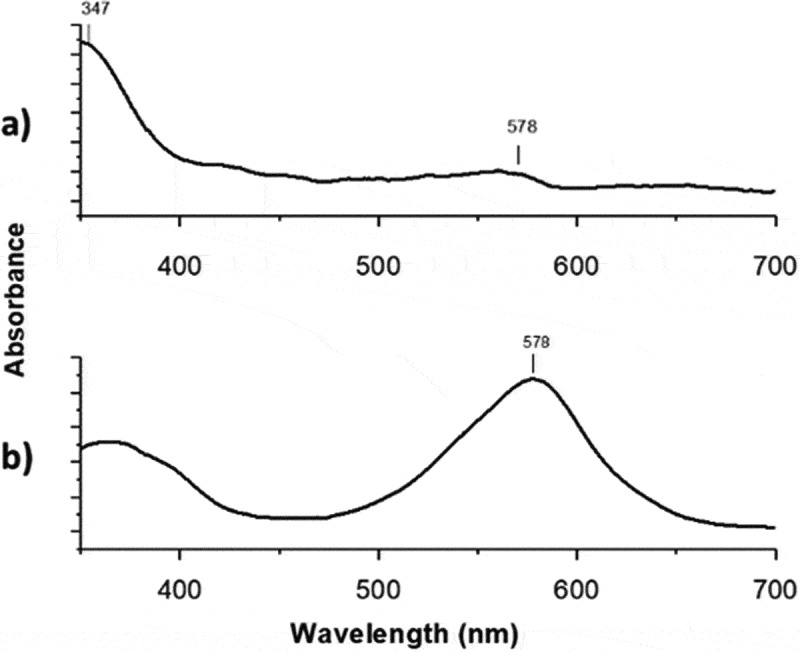
Absorption changes on the photoactive-polymer solution (3 g·L^−1^, in THF) a) before irradiated and b) after irradiated at 365 nm

Figure 3 shows the effect of irradiating the films at 365 nm on the absorption spectrum. Before UV irradiation (Figure 3a) the film shows an absorption at 347 nm. After irradiating, the photoactive film shows a maximum at 578 nm (Figure 3b) indicating their photoconversion from *SP* to *MC* (open conformation) [34,35].

### Fluorescence modulation by incorporating luminescent ZnS NPs in the photoactive polymer films

The photochemical and photophysical properties of the porous nanostructured systems can be changed by incorporating ZnS NPs, this effect is attributed to the overlapping of the electronic states between polymer and NPs [24]. In this system, their structural and electronic modifications, due to the interplay of photochromic components, can be studied.

In principle, ultraviolet irradiation generates the corresponding isomerization to merocyanine (*MC*) with an absorption band at 578 nm. The *MC* form can be attached to the surface of the inorganic ZnS NPs through the nitrogen and oxygen of the iminium and nitro groups, respectively. The emission of the inorganic NPs can overlap those of the *MC* and can be transferred to the photogenerated state of the photochromic component returning them to the closed-ring isomerization step (spiropyran ring, *SP*). Thus, this transformation can encourage the transfer of one electron from the excited inorganic NPs to the photogenerated state of the photochrome. Consequently, due to the transformation of the photochromic ligands upon ultraviolet irradiation, the emission spectrum of the nanostructured system shows a pronounced decrease in the luminescence. The switch of MC moiety into SP should disrupt the interaction between the polymer and ZnS NPs.

**Figure 4.**
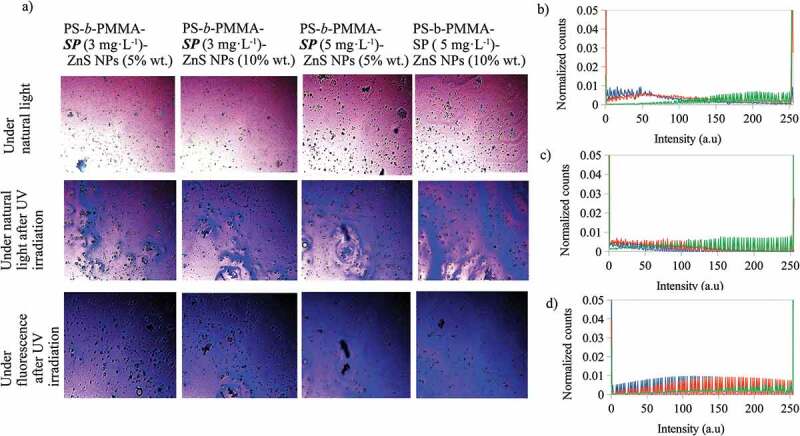
A) Optical microscope images at 20 X for the photoactive polymer with ZnS NPs (in CS_2_) under natural light, and under natural light and fluorescence, after UV irradiation at λ = 365 nm. Normalized counts for the total intensity of the channels b) red (R) c) green (G) and d) blue (B) of the images for the PS-*b*-PMMA-*SP* films with ZnS NPs prepared from 3 g·L^−1^ with ZnS NPs at 5% wt, – under natural light, – under natural light after UV irradiation at λ = 365 nm and – under fluorescence after UV irradiation at λ = 365 nm

**Figure 5.**
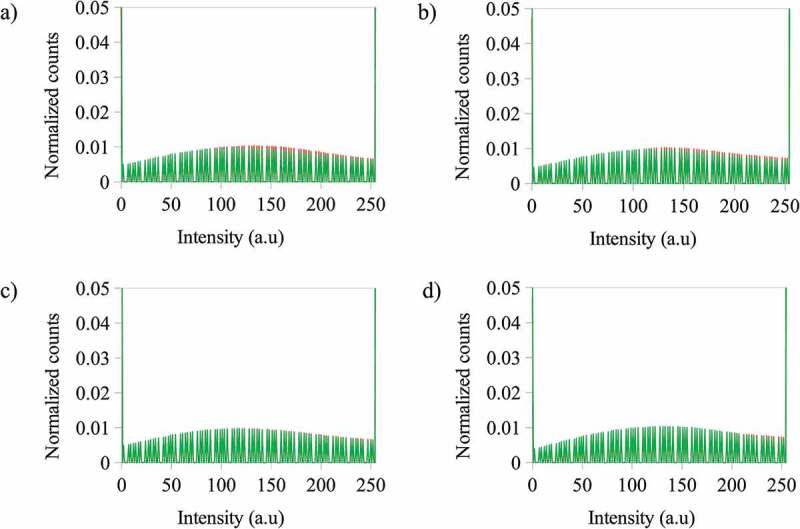
Normalized counts for the total intensity of the blue (B) channel for the images of the photoactive polymer (in CS_2_) under fluorescence after irradiation at λ = 365 nm at a) – 3 g·L^−1^ with ZnS NPs at 5% wt and – 5 g·L^−1^ with ZnS NPs at 5% wt b) – 3 g·L^−1^ with ZnS NPs at 10% wt and – 5 g·L^−1^ with ZnS NPs at 10% wt c) – 3 g·L^−1^ with ZnS NPs at 5% wt and – 3 g·L^−1^ with ZnS NPs at 10% wt d) – 5 g·L^−1^ with ZnS NPs at 5% wt and – 5 g·L^−1^ with ZnS NPs at 10% wt

**Figure 6.**
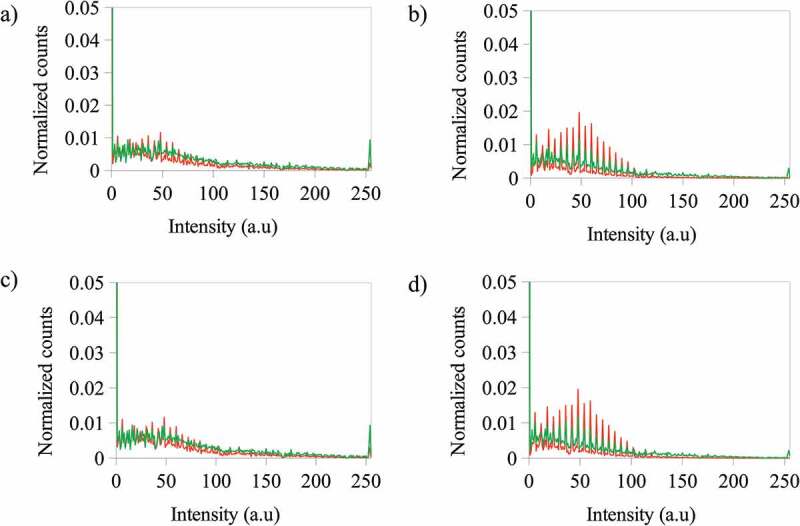
Normalized counts for the total intensity of the red (R) channel for the images of the photoactive polymer (in CS_2_) under fluorescence after irradiation at λ = 365 nm at a) – 3 g·L^−1^ with ZnS NPs at 5% wt and – 5 g·L^−1^ with ZnS NPs at 5% wt b) – 3 g·L^−1^ with ZnS NPs at 10% wt and – 5 g·L^−1^ with ZnS NPs at 10% wt c) – 3 g·L^−1^ with ZnS NPs at 5% wt and – 3 g·L^−1^ with ZnS NPs at 10% wt d) – 5 g·L^−1^ with ZnS NPs at 5% wt and – 5 g·L^−1^ with ZnS NPs at 10% wt

**Figure 7.**
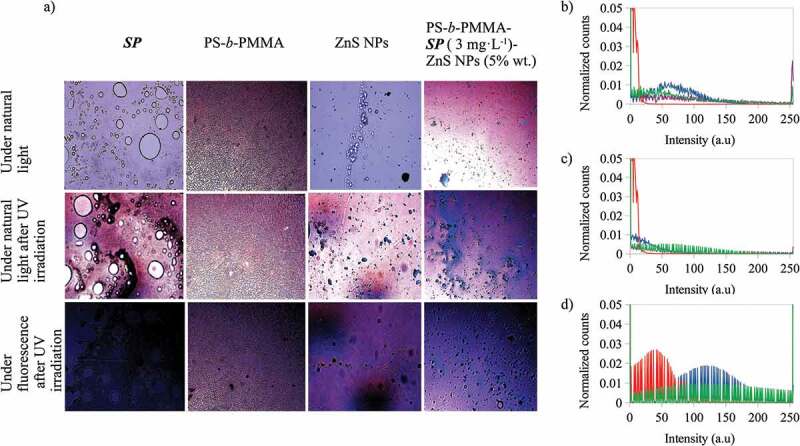
A) Optical microscope images at 20 X for the 1-(2-hydroxyethyl)-3,3-dimethyl indoline-6-nitrobenzopyran (*SP*), PS-*b*-PMMA-*SP*, ZnS NPs and PS-*b*-PMMA-*SP* photoactive polymer with ZnS NPs (in CS_2_) under natural light and under natural light and under fluorescence after UV irradiation at λ = 365 nm. Normalized counts for the total intensity of the channels b) red (R) and d) blue (B) of the images, after UV irradiation at λ = 365 nm, for a) – 1-(2-hydroxyethyl)-3,3-dimethyl indoline-6-nitrobenzopyran (*SP*) b) – PS-*b*-PMMA-*SP* c) – ZnS NPs and d) – photoactive polymer with ZnS NPs prepared from 3 g·L^−1^ with ZnS NPs at 5% wt

Moreover, the colors observed in the optical microscope images of the photoactive polymer-ZnS NPs, before and after irradiation (Figure 4), are the result of contributions of light emissions from all the components present in every composite material. The image colors could be represented in the RGB color space. Using color space, each pixel (color) of an image is described by a vector of three color components: red (R), green (G) and blue (B) [36]. R, G and B can take values in the range between 0 and 255 [36]. Then, the image is represented in RGB color space and the number of pixels (counts) for each value (intensity) of R, G and B can be separated red, green and blue colors and represented by histograms, thus, color histograms are obtained [37]. In this study, we have used the R, G and B color histograms to analyze qualitatively the contributions of the photoactive compound, the nanoparticle and their interactions, the effect upon the color changes in the images of the photoactive polymer-ZnS NPs (before and after irradiation). For this purpose, the histograms of the red (R), green (G) and blue (B) color channels of the optical microscope images of the composites were obtained using the software Fiji [38] (An open-source platform for biological-image analysis) with the plugin Color Histogram [39]. Every color histogram shows the number of pixels (counts) for each intensity value in the range from 0 to 255. To compare the obtained histograms, they were normalized dividing the counts by their total counts for the corresponding color channel. The main images and their color histograms are presented in Figure 4 to 7.

Figure 4 shows the optical microscope images for the photoactive polymer with ZnS NPs at different concentrations under natural light, and under natural light and fluorescence, after UV irradiation at λ = 365 nm. After UV irradiation, all composite colors are violet and blue and, under fluorescent light the blue color is enhanced. Additionally, the blue color intensity increases when the photoactive polymer concentration increases from 3 to 5 g·L^−1^ with ZnS NPs at the same concentration (5 or 10% wt). Also, the highest blue intensity was observed for photoactive polymer at 5 g·L^−1^ and with ZnS NPs at 10% wt. To determine the relationship of the color image with the R, G and B color channels, the histograms are shown in Figure 4 (b, c, d) for photoactive polymer at 3 g·L^−1^ with ZnS NPs at 5% wt. The histograms for the other composites are similar to that presented in Figure 4. As can be noted, the R and B channels change after UV irradiation. For the red channel, the signal intensity decreases but, for the blue channel, the number of counts increases. Although the intensity for the green channel decreases, it is smaller than for blue and red channels. Therefore, it could be inferred that the change of the color composites after irradiation could be mainly related to the changes of the R and B channels.

Also, it was found that when the photoactive polymer concentration increases from 3 to 5 g·L^−1^ with ZnS NPs at the same concentration (5 or 10% wt), the count of pixels for the blue channel slightly increases for intensities greater than 100 a.u. However, at the same photoactive polymer concentration, the distribution of the counts of the pixels is not changed with increasing of ZnS NPs concentration (see Figure 5). The blue color mentioned in this context is a component of the emission registered with the fluorescence microscope.

The behavior of the red channel differs from that of the blue channel (see Figure 6). In this case, when the photoactive polymer concentration increases from 3 to 5 g·L^−1^ with ZnS NPs at 5% wt, the change of the count of pixels for the blue channel is not significant, but at 10% wt the counts for 3 g·L^−1^ is lower than for 5 g·L^−1^. The same behavior is present when the NPs concentration is increased at constant photoactive polymer concentration.

To explain the effects of the components on the color emissions of the composite films, Figure 7 presents the optical microscope images of components SP, PS-b-PMMA, ZnS NPs and PS-b-PMMA-SP photoactive polymer (3 g·L^−1^) with ZnS NPs (5% wt) under natural light and under natural light and fluorescence after UV irradiation at λ = 365 nm. Also, their red, green and blue color histograms are presented under fluorescence after UV irradiation at λ = 365 nm. It was observed that, after UV irradiation under fluorescence, SP emits a dark blue color, PS-b-PMMA a violet color and ZnS NPs emits violet and blue colors. The histograms for the red and green channels of the composite are more similar to that presented by the polymer than for the other components, however for the blue channel the composite signal is greater than for the polymer and smaller than for the ZnS NPs and SP. Meaning that, for the composite the SP to MC, conversion and ZnS NPs are the main contributors to the blue color emission, and ZnS NPs and the polymer for the violet color.

### Characterization by fluorescence optical microscope and SEM

**Figure 8.**
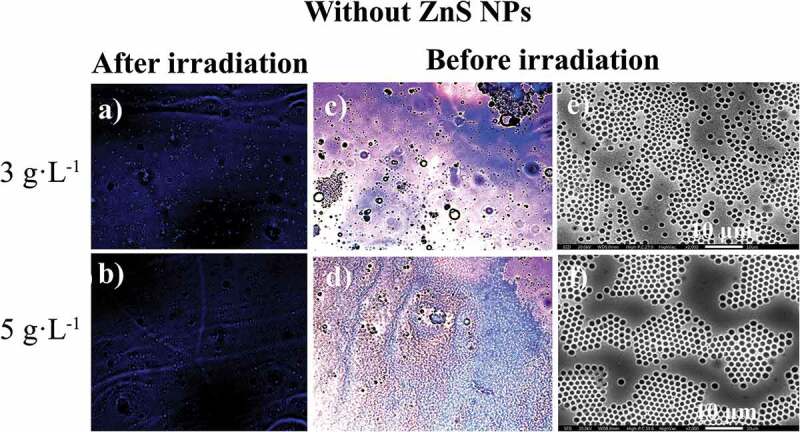
Optical microscope (in CS_2_) images for the photoactive PS-*b*-PMMA-*SP* functionalized without ZnS NPs after irradiation under fluorescence (a, b) at 3 g·L^−1^ and 5 g·L^−1^, and before irradiation (c, d) and SEM (in THF) images (e, f)

**Figure 9.**
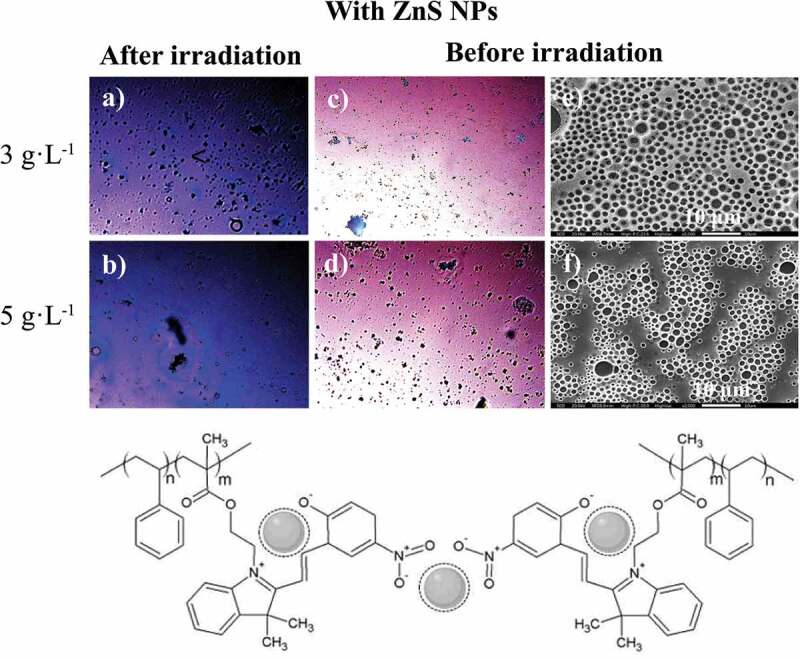
Optical microscope (in CS_2_) images for the photoactive PS-*b*-PMMA-*SP* functionalized with ZnS NPs after irradiation under fluorescence (a, b) at 3 g·L^−1^ and 5 g·L^−1^, and before irradiation (c, d) and SEM (in THF) images (e, f)

Determination of the photoluminescence properties of both components of the photoactive polymer is crucial to understanding their mutual roles within the self-assembled system. For this purpose, the optical images of the surface of PS-*b*-PMMA-*SP*, at 3 and 5 g·L^−1^, in absence and presence of ZnS NPs (5% wt) were compared under natural light and under fluorescence after UV irradiation, and also their SEM images under natural light before irradiation (see Figures 8 and 9).

The SEM micrograph of the films prepared with the absence of NPs (Figure 8 (e, f)) shows a certain array of pores on the surface. Condensed water droplets interact with the acrylic acid and SP moieties grafted to the polymer backbone leading to a porous organized surface. It is expected that an interaction between the ZnS NPs and the SP moiety alters the aforementioned array.

Figure 9, shows the films prepared in the presence of ZnS NPs. There is a loss observed with respect to the honeycomb arrangements when compared with the film surface absence of NPs. This effect suggests an interaction between the NPs and the SP moiety. Consequently, the SPs are no longer available to interact with the condensed water droplets because of the presence of the NPs. Thus, the process which drives and organizes pore distribution in the whole surface is changed (Figure 8 (e, f)). In general, the film surfaces and their porous distributions are governed by thermodynamic factors. The increase in interfacial tension (restricted polymer-chain mobility due to the coordinated ZnS NPs) leads to a more highly disordered porous polymer film.

It was determined that in the absence of the NPs, the color emission was dark blue as shown in Figure 8 (a, b). The incorporation of the ZnS NPs into the PS-b-PMAA*-SP* promotes the blue and violet color emissions after UV irradiation (Figure 9 (a, b)). The blue luminescence observed was attributed to the conversion of the photochromic agent incorporated in the polymer matrix, specifically from the interaction between the *SP* to *MC* transition and the ZnS NPs when are present. The resultant SEM images suggested that in the presence of the ZnS NPs, a segregation of the PMMA-*SP* functionalized block is caused, which affects the interaction with the condensed water droplets, which drive the pore formation (Figure 9 (e, f)).

## Conclusions

The PS-*b*-PMMA was grafted with the *SP* component and subsequently functionalized with ZnS NPs yielding stimulus-responsive porous surface films. The porous films exhibited light sensitive behavior under UV-light irradiation procedures. Moreover, the optical and morphological properties changed under UV light irradiation. The incorporation of the ZnS NPs in the photoactive polymer promoted blue and violet color emission after UV irradiation. Therefore, the blue luminescence was attributed to the conversion of *SP* to *MC* in the polymer matrix interacting with the ZnS NPs. The results of scanning electron microscopy (SEM) showed that a low arrangement of pores occurs in the presence of NPs. The NPs cause unfavorable conditions for the hexagonal film formation using the BF method. These materials could potentially be very attractive for their use in the manufacture of light-sensitive porous films due to their optical response.
